# Effective Area Measurements of Magnetic Pick-Up Coil Sensors for RFX-mod2

**DOI:** 10.3390/s22249767

**Published:** 2022-12-13

**Authors:** Domenico Abate, Roberto Cavazzana

**Affiliations:** Consorzio RFX (CNR, ENEA, INFN, Università di Padova, Acciaierie Venete SpA), C.so Stati Uniti 4, 35127 Padova, Italy

**Keywords:** magnetic diagnostics, pick-up coils, calibration

## Abstract

A meaningful characterization of the magnetic configuration of toroidal plasmas requires the identification and estimation of the sources of error on each magnetic measurement of the overall diagnostic system. Thus, the correct characterization of magnetic pick-up coil sensors and the assessment of their reliability becomes a necessary requirement before their permanent installation in the RFX-mod2 experiment. The experimental characterization methodology developed for the three-axes magnetic pick-up coil sensors of RFX-mod2 experiment is presented here. The sensitivity of each sensor is evaluated not only by performing accurate measurements of the effective areas in a time-varying magnetic field, but also by checking the alignment of the magnetic axes through measurements of the effective areas at different rotation angles. Moreover, the effect of thermal cycles on measuring the effective area and the angle of misalignment are evaluated and analyzed.

## 1. Introduction

The RFX-mod2 experiment is a major upgrade of the reversed field pinch (RFP) magnetic confinement experiment RFX-mod with the aim of improving the control of magnetic confinement, plasma density, and plasma wall interaction in both RFP and Tokamak configuration [1,2], with plasma pulse duration up to 5 s. The upgrade consists in the removal of the highly resistive vacuum vessel, so that the copper passive stabilizing shell (PSS) will be the conducting structure nearest to the plasma. In particular, the PSS will be deeply modified in order to operate in vacuum conditions sustaining the new first wall and the wide system of in-vessel diagnostics.

The RFP configuration is characterized by the excitation of a wide spectrum of magnetohydrodynamic modes during the plasma discharge; therefore, a high number of sensors is required in both toroidal and poloidal directions in order to obtain a sufficient spatial resolution to obtain a correct reconstruction of the plasma magnetic field structure [3]. Moreover, the proper characterization of fast MHD events, such as magnetic reconnections [4,5], requires a useful bandwidth of at least 100 kHz. The general constraint of maximizing the signal to noise ratio always holds in order to achieve a high quality of the acquired signal. Finally, other technological constraints are present: intense magnetic fields and flux variations, high temperature resistance, and vacuum compatibility. Given technical requirements and constraints, sensors based on pick-up coils had been deemed as the most suitable in this context.

A necessary condition to properly characterize the plasma magnetic configuration is to identify and estimate the sources of error on each magnetic measurement. In this paper, a detailed experimental characterization of the magnetic pick-up coil sensors of RFX-mod2 experiment is presented. The assessment of their reliability is a necessary requirement before their permanent installation in the experiment. An experimental characterization methodology is developed and presented for both three-axes and two-axes magnetic pick-up coil sensors of RFX-mod2 experiment. The sensitivity of each sensor is evaluated not only by performing accurate measurements of the effective areas in a time varying magnetic field [6,7,8,9,10], but also by checking the alignment of the magnetic axes through measurements of the effective areas at different rotation angles. The effect of thermal cycles on both the effective area and the angle of misalignment are evaluated and analyzed.

## 2. The RFX-mod2 Magnetic Pick-Up Coils

The pick-up coils are magnetic sensors made of a small coil of wire able to measure the magnetic field in the vicinity of a point in space. In principle, in a uniform magnetic field, varying with time B(t), the voltage induced in the coil is:

(1) $$V\left(t\right)=A_{s}\;\frac{dB(t)}{dt}$$

where $A_{s}=\sum_{k=1}^{N}A_{k}$ is the collecting area of a sensor with *N* turns, being the sum of the areas $A_{k}$ of each single loop. The nominal value of $A_{s}$, usually termed as NA in a loose sense, can be defined by a proper design. A signal proportional to the magnetic field B(t) of a pulsed experiment can be then obtained by an analog or digital integration. The sensor design is a trade-off among different constraints. From an electrical point of view our aim was to maximize the sensitivity by increasing the effective area $A_{s}$—and therefore the signal to noise ratio—while reducing the stray inductance of each axis and the parasitic coupling between them in order to preserve a significant bandwidth.

The pick-up coil sensors are the basic diagnostic for the investigation of several physical phenomena in RFX-mod2: the accurate reconstruction of MHD spectrum, the investigation of magnetic turbulence physics, the detection of fast particles and the investigation of electromagnetic forces. These requirements are quantitatively and qualitatively different as the magnetic configuration changes (i.e., tokamak or RFP) because of the very different levels of plasma current that can be performed in RFX-mod2 (e.g., 100 kA in tokamak and 2 MA in RFP). Thus, four different types of pick-up coils have been designed:

- 3AX: three-axes sensor for measuring all the magnetic field components (toroidal, poloidal, radial) with a bandwidth up to 200 kHz.
- 2AXH: two-axes sensor for measuring both toroidal and high frequency poloidal component with a bandwidth up to 2 MHz.
- 1AX: single-axis sensor for the measurement of the toroidal magnetic field with a bandwidth up to 200 kHz.
- 2AXE: two-axes sensor for measuring the toroidal and poloidal components of the magnetic field outside the shell with a bandwidth up to 200 kHz.

**Figure 1:**
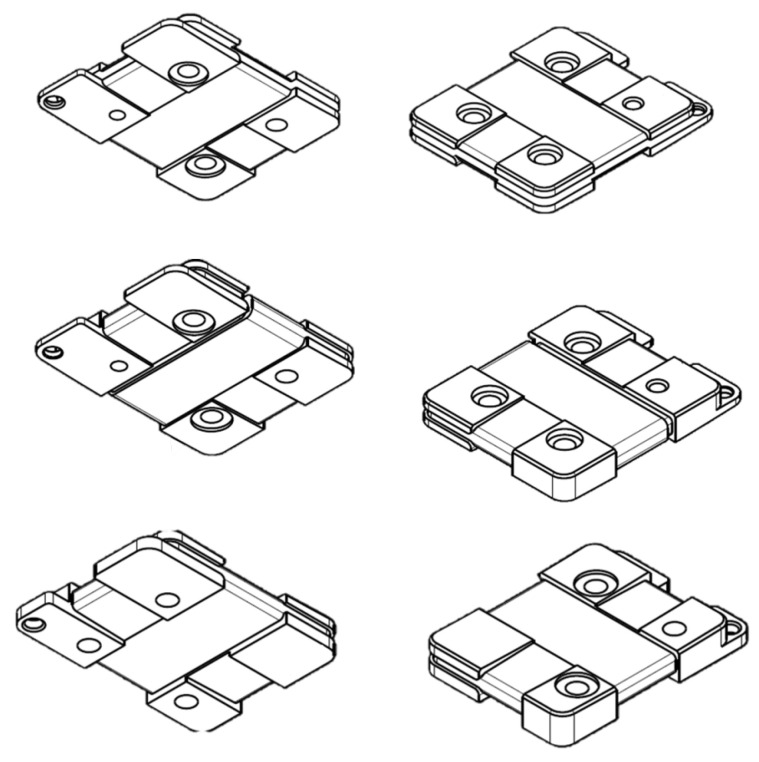
Three axes reel (**top**), two axes and single axes reel (**middle**), external two axes reel (**bottom**).

**Figure 2:**
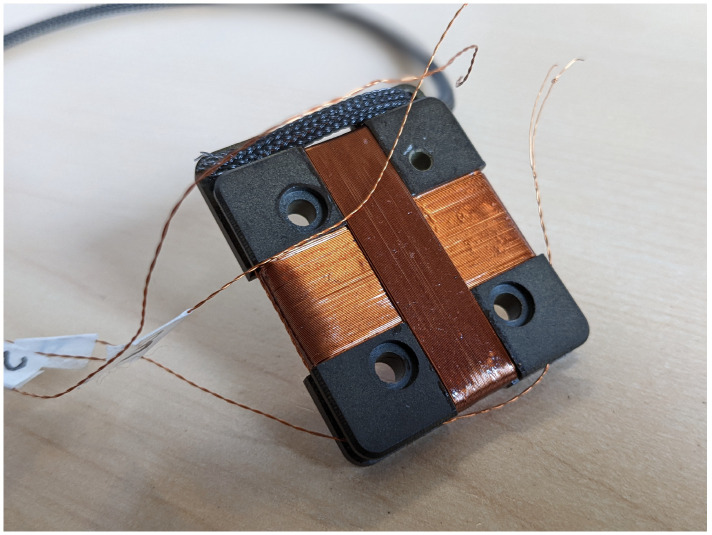
Three axes (3AX) pick-up coil sensor prototype.

The four types of sensors are designed as a direct evolution of the previous version of RFX-mod experiment [11]. The 3AX pick-up coil consists in three independent and orthogonal enameled copper windings on the same core (i.e., the reel) of about 200 ${\mathrm{mm}}^{2}$ of cross-section surface [12]. Because the sensors will be installed in a vacuum environment, all the materials must be vacuum compatible. Moreover, the sensors will be placed in the internal and external surfaces of the PSS which will undergo thermal cycles during baking treatments and transients during the plasma operations, with temperatures up to 200 °C. Therefore, the core is manufactured in glass fiber reinforced polyamide-imide (Duratron PAI 5530) which is characterized by high thermal stability and a thermal expansion coefficient close to the copper one. The adopted enameled copper wire is coated with polyamide-imide with a maximum operating temperature of 210 °C. Each type of pick-up is characterized by a different layout of the core that houses the winding as shown in Figure 1: the internal sensors share the same support of 47×37.7×6 mm while the external ones have a different one of 47×34.2×6.5 mm. This difference was required in order to avoid interference with other parts of the PSS supporting structure. An important requirement is to keep the windings of each pick-up fixed in such a way that the effective area is not affected by the thermal cycles of the plasma discharge operations. Because the use of any adhesive tape is not allowed in vacuum, each winding was treated with a paint impregnation process to ensure the stability of each effective area. The adopted low-viscosity two-component epoxy resin polymerizes at room temperature and has a maximum operating temperature of 260 °C. The pair of wires exiting from each winding has been twisted with a maximum lay length of 5 mm in order to prevent the concatenation of additional magnetic flux density by spreading the effective area. All the twisted pairs of wires have been protected by collecting them inside a sheath made of Flexo PPS Polyphenylene sulfide. The pick-up coils are fastened behind the graphite tiles directly on the PSS by two threaded rivets made of PEEK 450G ensuring both electro-mechanical and vacuum requirements. The side of the reel facing the PSS is carefully machined to fit the poloidal curvature in order to minimize the radial size and position misalignment. The two rivets pass through the two counterbores on the side facing the PSS; these are realized in order to house the silicone rubber washers needed for electrical insulation [13]. The end part of the rivets are threaded for easier installation of the sensors on the PSS by using an hexagonal nut made of PEEK. Finally, the nut and the rivet are welded together by an ultrasonic plastic welding process. All the materials have been verified for vacuum compatibility with an atmospheric thermo-gravimetric analysis consisting in two thermal cycles of 1 h each at the temperature of 200 °C: all the materials revealed a mass loss largely below the 1% suggesting their vacuum compatibility [14]. A picture of the 3AX sensor prototype is shown in Figure 2.

The minimum magnetic field required to be measured is 1 mT/s corresponding to the number of turns and layers for each winding as given in Table 1. The geometric area is computed as the surface defined by a single turn times the number of turns. The self and mutual inductance values for each pick-up coil sensor have been estimated by modeling the real 3D geometry using FastHenry; the results are summarized in Table 1. The measurements performed on the pick-up prototypes using a LCR meter revealed a relative percentage error below ±5%.

## 3. Effective Area Measurements

**Figure 3:**
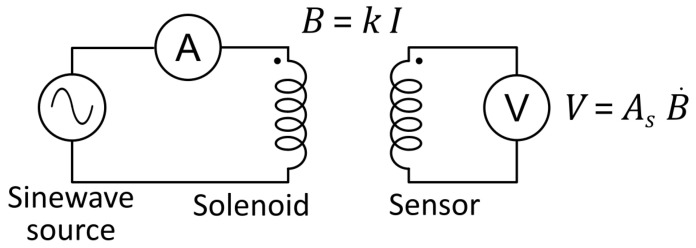
Schematic of the basic principle of the measure.

The nominal value of $A_{s}$ defined by the design, is inevitably subjected to variations, mainly due to the manufacturing process and subsequent thermal cycles during operation. Even if the associated errors due to this processes can be contained in a reasonable range (say within 5%), in the case of fusion relevant experiments each single measurement requires a nominal error of around 1% [15]. Obviously this goal can be achieved only by an individual calibration for each sensor. The typical straightforward setup used to this purpose is shown in Figure 3.

Since in the frequency domain (1) reads as:

(2) $$V\left(\omega \right)=A_{s}\;j\omega B\left(\omega \right)$$

in principle, the effective area $A_{s}$ characteristics of a magnetic pick-up coil sensor can be extracted from measurements performed in a known sinusoidal time varying magnetic field, obtained by a straight “long” solenoid [6,8,9,16] or an Helmoltz coil setup [17]. The solenoid has to be sufficiently large to produce a reasonably uniform field in the volume pertaining the sensor. So, given the geometrical factor of the coil system *k* and the excitation frequency of the sine-wave source f=ω/2π:

(3) $$A_{s}=\frac{V(f)}{2\pi f\;kI(f)}$$

The application of this straightforward scheme in the experimental practice brings a series of subtleties which are discussed in following sections.

## 4. Impact of Solenoid Geometry on the Reference Magnetic Field

**Figure 4:**
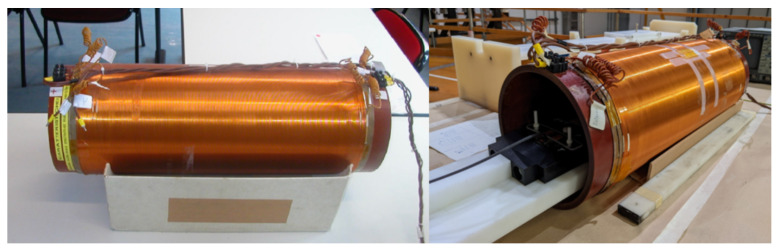
Finite rectilinear uniform solenoid adopted as source of reference magnetic field.

The reference sinusoidal magnetic field has to be known with a high level of accuracy. Thus, a fully characterized finite uniform rectilinear solenoid (Figure 4) is adopted as source of the reference magnetic field [18]. The nominal values of the solenoid are reported in Table 2, where the on-axis magnetic field for 1A of current is evaluated at half of the length (L/2) and the electrical parameters are obtained by using an impedance analyzer (*HP 4194A*) in the frequency range of 100 Hz–10 kHz.

Despite the accuracy in modeling and manufacturing the solenoid, a possible source of error—which has not been considered in the past—is related to the impact of small variations of the solenoid geometry on the produced magnetic field. These correspond to the possible error of measurement of the solenoid dimensions. In particular, three cases have been considered and analyzed:

- Impact of variations in the length (*L*) of about ±0.5 mm;
- Impact of variations in the radius (*r*) of about ±0.25 mm;
- Impact of the elliptical cross section (rather then perfectly circular) with a measured eccentricity (*e*) of about 0.07; this corresponds to an error of 0.28 mm on one axis of the ellipse if the other would be at the nominal radius value.

**Figure 5:**
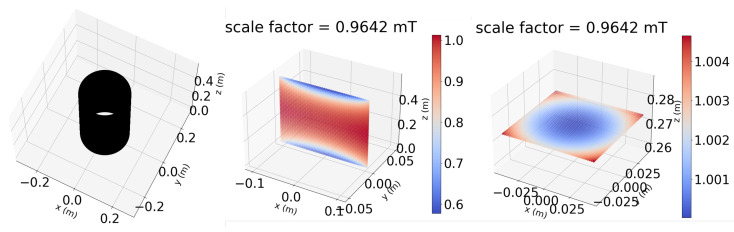
Geometry of the solenoid (**left**), magnetic field on the xz plane (**center**) and magnetic field on the xy plane (**right**).

For each case, the on-axis magnetic field is evaluated both numerically (using the Biot–Savart law) and analytically [19]. Then, the relative percentage error with respect to the reference case (nominal geometry) is computed. As reported in Table 3, the impact of variations on both length or radius are always below one part per thousand (particularly 0.08%) while the error due to eccentricity is an order of magnitude lower, about two parts per ten thousand (0.02%). The spatial distribution of the magnetic field is shown in Figure 5 for the reference nominal geometry case.

## 5. Electrical Measurements

### 5.1. Limitations of Basic Methods

A common implementation of the AC measurements for the scheme of Figure 3 can be realized using standard precision multimeters and an signal generator combined with an audio amplifier. Using a setup such as this, the effective area of the sensor $A_{s}$ can be measured as:

(4) $$A_{s}=\frac{V_{rms}}{2\pi fkI_{rms}}$$

However this method introduces some limitations, which are difficult to evaluate and control. First, this method is sensitive to the harmonic distortions of the output stage driving the solenoid. Even considering a low harmonic content, the implicit derivative amplifies by a factor *n* the amplitude of each *n*-th harmonic, which add up in the $V_{rms}$ measurement. For example a mere 0.1% (−60 dB) on the 5th harmonic would introduce in the ratio $V_{rms}/I_{rms}$ a sensible 0.4% error.

The second issue is related to instrument noise and the EMI coming from the environment, which add up in the $V_{rms}$ measurement of the coil, being a low voltage measurement.

A third subtle issue—found during preliminary test—was a noticeable gain drift of the audio amplifier when frequency or amplitude are changed, and which took several minutes to stabilize within a degree compatible with the precision of the measurement chain. Its impact on the measurements can be mitigated by taking the current and voltage measurement in the same time interval.

### 5.2. Electrical Measurement Setup

In order to overcome the aforementioned troubles, it has been adopted a multichannel acquisition (DAQ) system (*Yokogawa WE 7000* - 1 MSamples/s, 14 bit) to synchronously acquiring the signals of the coil current and the voltages of each coil of the sensor. Moreover, this system allows to measure several sensors simultaneously, speeding up the process in preparation for handling the high number (about one thousand) of RFX-mod2 sensors to be calibrated.

**Figure 6:**
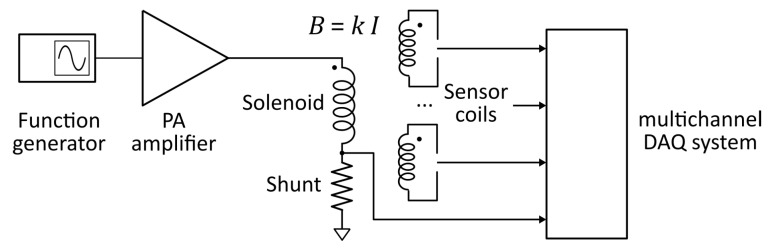
Realized electrical measurement setup.

The schematic layout of the system is shown in Figure 6. A programmable wave generator (*SIGLENT SDG2042X*) drives a public addressing (PA) amplifier (*PASO 4010*). The PA amplifiers are designed to handle a range of impedance loads (2.5–100 Ω) significantly wider than conventional audio amplifiers (usually 4–16 Ω).

Given the characteristics value of $A_{s}$ of the probes, the solenoid constant *k* and the range of the bench instruments (maximum current range 2 A, lowest voltage range 100 mV), the usable frequencies fall in range between 200 Hz and 1000 Hz, which is the range at which the parasitic effects on the solenoid can be neglected.

**Figure 7:**
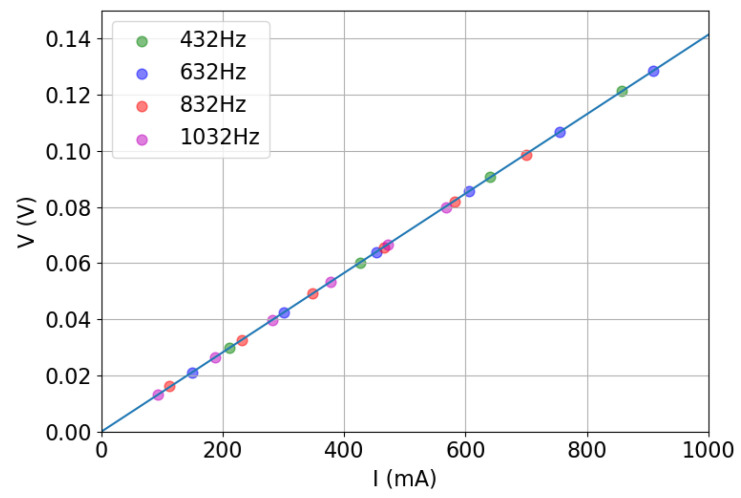
V-I relation for the shunt calibration with the multimeter.

The resistive shunt (Hobut SHR2A200, 2 A, 200 mV, 0.1 Ω) has been calibrated using the current measurement provided by the digital multimeter (*HP 3474*): a linear regression fit of the relation between the voltage measured over the shunt by the DAQ system and the current given by the multimeter allows to verify that an accurate measurement has been achieved (Figure 7). In fact, the slope of the linear fit provides the value of the electrical resistance of the shunt with an error below the 0.03%; the intercept is related to the systematic error due to the noise present on the chain of measurement which is extremely low (Table 4).

The effective area measurements were performed at different frequencies and input voltages for the sinusoidal wave generator. The exact frequencies used were 232 Hz, 432 Hz, 632 Hz, and 832 Hz, in order to reduce the effects of the harmonics disturbances coming from the AC mains at 50 Hz. The voltage amplitudes (about 50, 75, and 100 Vpp at the output of the PA amplifier) were in range suitable to avoid the saturation of the amplifier and to ensure a high enough level to provide a good signal to noise ratio on the measured signals of the probes.

### 5.3. Measurement of the Coil/Sensor Coupling at Fundamental Frequency Component

As mentioned before, a fitting method has been developed to replace of the $I_{rms}$ and $V_{rms}$ values of Equation (4) with the amplitudes $I_{0}$ and $V_{0}$ determined on the single fundamental component at frequency *f* of the measured signals, so that the effective area $A_{s}$ can be obtained with better confidence. For this purpose we use a nonlinear fitting least square algorithm (NLSQ fit) [20] to fit the acquired signals with the function:

(5) $$A\left(t\right)=A_{0}\cos\left(2\pi f\;t+\varphi \right)$$

where $A_{0}$, *f*, and φ are the three fitting parameters.

In order to allow the NLSQ algorithm to work correctly for this specific case, even with very noisy signals, it has to be initialized with a decent guess of the parameters, along with the offset and large disturbance removed from the acquired data points. The fitting is performed in four steps:

1. The first guess for amplitude $A_{0}$, frequency *f* and phase φ are determined using the peak amplitude from an FFT of the raw sampled signal.
2. The offset is roughly removed by subtracting the mean value of the raw data. The first step of the NLSQ fit is used to determine more precisely the fundamental frequency.
3. The offset bias due to the acquisition windowing of the sinusoidal component is then removed by selecting the raw data points to match exactly an integer number of periods, using the frequency found in the previous step. The offset is removed as a mean value again and the second pass of the NLSQ fit is applied.
4. Finally, the values of the raw data points which have a deviation larger than 2.5 σ from the previous fit are discarded, removing EMI spurious interferences. This further improves the residual offset removal and gives a more robust convergence of the LSQ fit. Then the third and final pass of the NLSQ fit is applied, giving the final estimate of the quantities.

**Figure 8:**
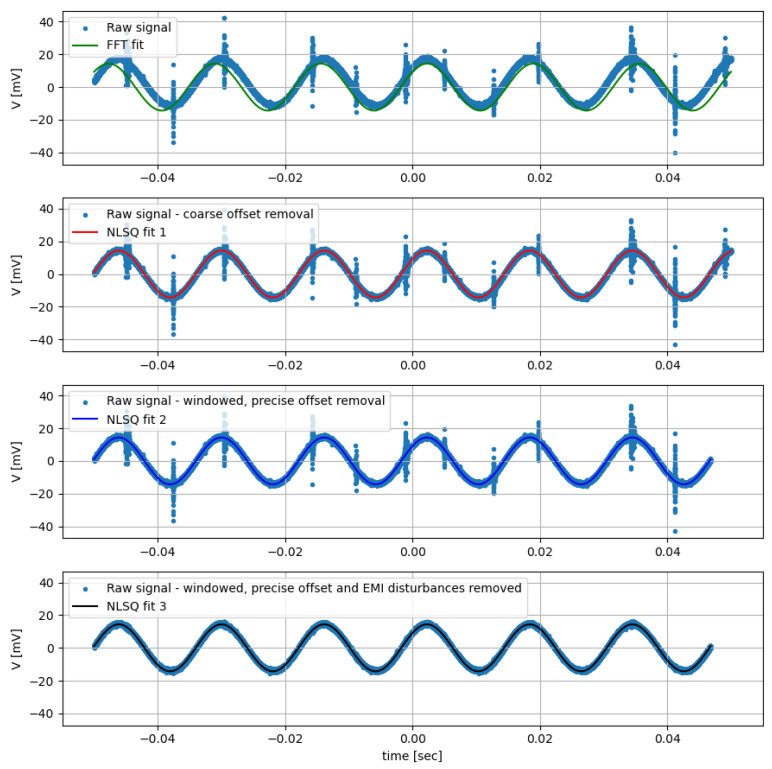
Example of the four steps used to recover the fundamental amplitude from an acquired signal.

The fit returns the optimal values for the parameters so that the sum of the squared residuals is minimized and the estimated covariance matrix from which the standard deviation σ for each parameter can be derived. For a low-frequency test example shown in Figure 8, the values found by the final fit are reported in Table 5.

It is worth mentioning the reason behind this somehow cumbersome method used to remove the DC offset, since in principle it could be avoided by simply using a fitting function, including the additional constant term representing the DC component. However, for this specific case the NLSQ algorithm with this additional degree of freedom does not behave properly, giving larger uncertainty and sometimes being affected by phase flipping and inaccurate amplitude results, especially with noisy signals.

## 6. Probe Calibration Set-Up

**Figure 9:**
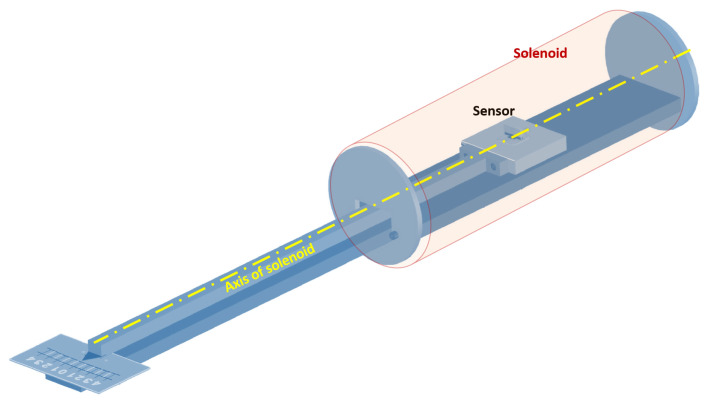
Representation of the support for the measurements performed at different angles of rotation.

The experimental set-up consists in a long uniform solenoid (Figure 4) providing a reference sinusoidal magnetic field. The pick-up sensor is fixed on a support placed inside the solenoid at its center (Figure 9) and machined with a tolerance of 0.05 mm. The support is fixed to a rotating rod allowing to define angles of rotation between the solenoid and sensor axes from $\pm 0.1^{\circ }$ to $\pm 6^{\circ }$.

In order to avoid magnetic field distortions, the solenoid was placed at the center of a large wood table and the fasteners of the table made of non-magnetic materials (Aluminum and AISI 316). The whole setup was located upon the wooden mezzanine floor of RFX experimental hall and the instruments kept at least a 2 meter distance from the solenoid.

The straight solenoid is sufficiently large and long to produce a reasonably uniform field in the volume pertaining the sensor. Here, we use a solenoid with an effective transducer constant $k=B/I=0.96437\pm 2\cdot 10^{-4}$ mT/A, which takes into account the field averaged in the volume of the sensor.

The sinusoidal current *I* flowing in the primary circuit is generated by a wave generator connected to an audio amplifier. This current produces the reference magnetic field *B* that crosses the section of the sensor. This varying magnetic flux induce a voltage between the two terminals of the sensor that is directly acquired using the multichannel DAQ system. In addition, a resistive shunt has been used for measuring the current. The scheme of the effective area measurement setup is shown in Figure 6.

### Variable Angle Measurement Procedure

**Figure 10:**
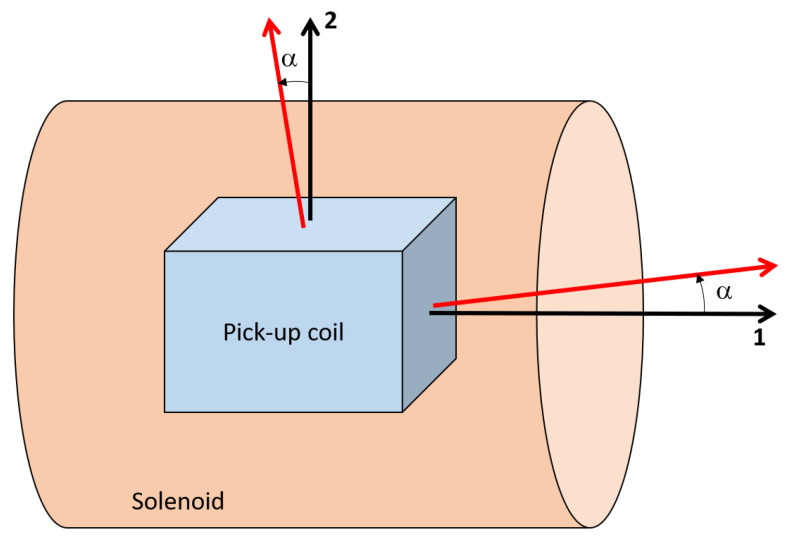
Graphic representation of the solenoid and the sensor with the related axes in black and red, respectively.

**Figure 11:**
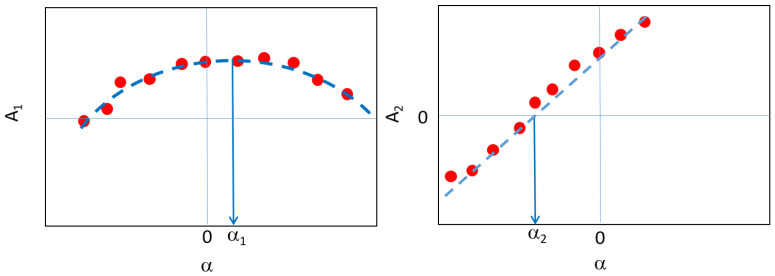
Representation of the effective area dependence with the rotation angle α for the two directions of measurements (ϕ, θ): the affective area and the angle of misalignment $\alpha_{0}$ are identified from best-fit of measurements.

It has to be remarked that for each winding of the sensor, the magnetic axis (i.e., the axis of measurement) does not necessarily correspond to the geometric axis of the reel. Such an angle of misalignment between geometric and magnetic axes has to be evaluated and quantified for each winding (i.e., direction of measurement). Considering the scheme in Figure 10, the primary direction (1) corresponds to the solenoid axis while the secondary direction (2) is the orthogonal one. The dependence of the effective area on the angle of rotation α is defined in (6) for both the directions of measurement and it is qualitatively represented in Figure 11. Thus, the angle of misalignment along the 1 direction (primary angle of misalignment $\alpha_{1}$) is determined by looking for the maximum value of the parabolic fit in (6). On the other hand, the orthogonal misalignment angle (secondary angle of misalignment $\alpha_{2}$) is determined as the value at which the linear relation in (6) crosses zero. Thus, both angles of misalignment are identified as a best-fit of the measurements performed at different values of the rotation angle. In the case under analysis, the two orthogonal directions of measurement are the toroidal and poloidal ones.

(6) $$\begin{array}{cc} A_{1} & =A_{1,0}\cos\left(\alpha \right)∼1-\frac{\alpha^{2}}{2}\\ A_{2} & =A_{2,0}\sin\left(\alpha \right)∼\alpha \end{array}$$

The measurement system in Figure 9 is a realization of the scheme in Figure 10 allowing to vary the angle α between the geometric axis of the sensor and the solenoid one.The effective area measurements are performed for different values of the rotation angle α, from $-6^{\circ }$ to $+6^{\circ }$ with increments of a degree; in the range of ±1 the increment is of $0.5^{\circ }$. For each angle position, multiple measurements in frequency are performed (432, 632 Hz) at wave generator voltage of 2 V. In the case of $\alpha =0^{\circ }$, the effective area measurements are performed at four values of frequency (232, 432, 632, 832 Hz) for each prescribed voltage of the wave generator (1, 1.5, 2 V). Thus, the variability with respect of frequency and voltage can be quantified. The voltage signals are acquired for both primary and secondary axes in the axial plane (1,2).

## 7. Results

**Figure 12:**
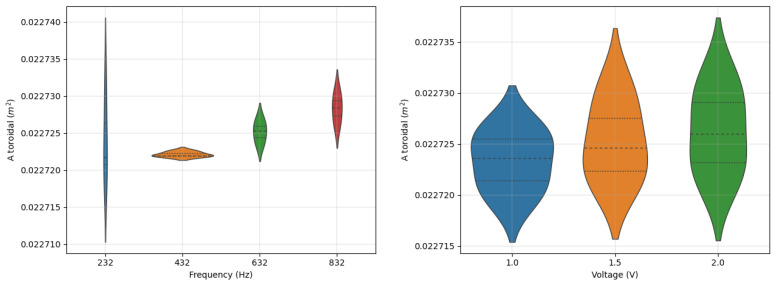
Violinplot for the effective area measurements of the 3AX toroidal winding as function of frequency (voltage variability) and voltage (frequency variability).

The effect of frequency and voltage can be quantified by performing multiple measurements: once the voltage (frequency) is fixed, the effective area is measured for different values of frequency (voltage). The measurements have been performed at different values of frequency (e.g., 232, 432, 632, 832 Hz) for different voltage levels of the wave generator (e.g., 1, 1.5, 2 V) providing a sample of measurements for each effective area. Then, the average and standard deviation are computed; thus, the error—commonly expressed in percentage—is defined as the ratio between the standard deviation and the average value. The error related to the frequency variability is extremely low for all the axes of measurement: for the 3AX sensors it has a maximum value of 0.05% while for the 2AXH and 2AXE about 0.1%. In the same way, the maximum error related to the voltage is about 0.05% for 3AX and 2AXE and 0.1% for 2AXH. The measurements performed on a 3AX toroidal winding are summarized in Figure 12, where it can also be noticed the symmetry of the sample measurements (dotted lines inside the violin indicating the quartiles).

**Figure 13:**
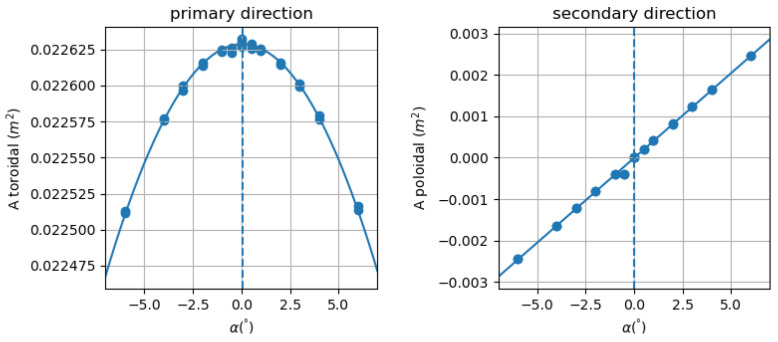
Toroidal (primary, left) and poloidal (secondary, right) effective area values as a function of the angle of rotation for the 3AX sensor; the angle of misalignment is represented by the vertical dashed line.

The misalignment angle of each axis of measurement has been quantified by performing multiple measurements at different angle of rotation and following the procedure of best-fit previously described. The results of the best-fit for the 3AX sensor is represented in Figure 13 where the primary and secondary misalignment angles are always below $0.2^{\circ }$. This corresponds to a percentage error following a cosine law (i.e., cos(α)−1) of about 0.0013%. It is important to analyze also the difference between the two misalignment angles ($\Delta \alpha =\alpha_{1}-\alpha_{2}$): this has to be as low as possible in order to ensure the orthogonality of the two magnetic axes. The measured levels of difference Δα are always below $0.3^{\circ }$ ensuring a satisfactory level of orthogonality.

A summary of the different sources of errors involved in measuring the effective area is reported in Table 6.

Finally, after the assessment of the presented measurement method, the effect of thermal cycles on measuring both the effective area and the angle of misalignment have been evaluated and quantified. The adopted thermal cycles are defined on the basis of RFX-mod2 baking expectations which can be summarized in slow transients and a maximum temperature of 200 °C. Each cycle is characterized by a rising temperature transient phase of about 40 min followed by a constant temperature phase of about 30 min; finally, the temperature is ramped down in about 30 min. These cycles have been executed on each sensor at increasing values of temperature (160 °C, 180 °C, 200 °C) for a total of 6 thermal cycles for each sensor. One sensor has been tested also at the temperature of 220 °C. The complete set of thermal cycles applied to each sensor is summarized in Table 7.

**Figure 14:**
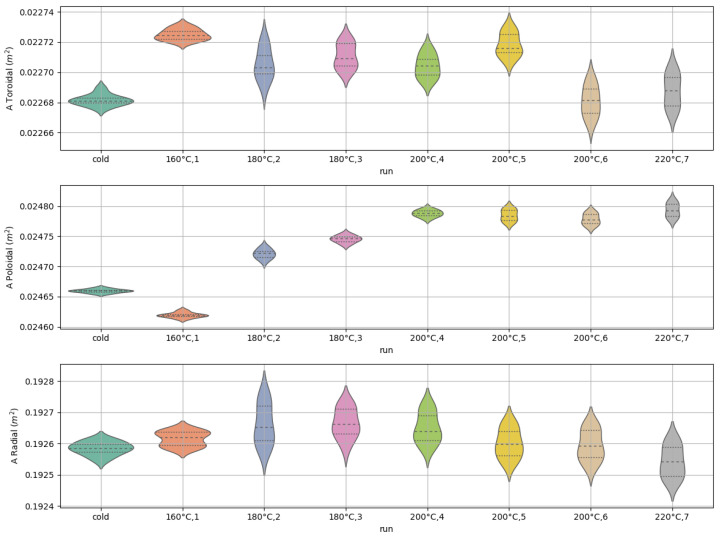
Effective area of each winding for the 3AXE prototype as a function of thermal cycles; the violin plot is obtained on different measurements performed in frequency and voltage.

The measurements revealed that the effective area is stable against thermal cycles of increasing temperature for all the radial winding (3AXC, 3AXE, 3AXF) and the majority of the toroidal one (3AXC, 3AXE). The area of the poloidal winding instead shows a clear tendency to slightly increase as a result of thermal cycles of a modest value about 0.7%. These results are summarized in Figure 14 for a single 3AX sensor prototype but similar results have been found for the other prototypes. An increment of the angle of misalignment is observed reaching maximum values of about $0.3^{\circ }$ but preserving the orthogonality between the two axes.

## 8. Summary and Conclusions

The characterization method for the magnetic pick-up coil sensors of RFX-mod2 experiment presented here provides a reliable and reproducible method to calibrate the effective area within 1% in a time varying magnetic field and to verify the correct alignment of the sensor coils. The procedure to determine the effective area has been developed by reducing at minimum all the relevant error sources found during a preliminary test phase carried out by direct measurements of solenoid current current and probe voltages with precision multimeters. Some of the issues found (EMI induced noise, harmonic distortion and gain stability of the amplifier) have been solved by replacing the multimeters with a multichannel DAQ system and by the application of appropriated signal post-processing. Another improvement has been obtained by using test frequencies which are not multiples of the 50 Hz grid frequency. Furthermore, the use of a multichannel system allows to perform the simultaneous characterization on up to 4 probes at the same time, in view of a significant reduction in the time needed to calibrate all the 724 sensors being installed. The final estimation of effective area of the sensors is determined by measuring the effective areas at different rotation angles with respect to the solenoid reference axis and performing a parabolic fit. In the case of multiple-axis sensors, the method is extended to check for the possible non-orthogonality between the couples of sensor coils by taking simultaneously the measurement of the “stray” area and estimating the relative orthogonal angle by means of a linear fit. Finally, the impact of multiple thermal cycles on the effective area have been evaluated, showing that they can be easily removed by performing a thermal preconditioning of each sensor before the final installation. 

## Figures and Tables

**Table 1:** Main features of each pick-up coil sensor including the geometric area: the radial, poloidal, and toroidal axes of measurements are denoted as *r*, *p*, and *t*, respectively.

Sensor	Axis	Turns	Layers	Diameter	Length	Geometric Area NA ($m^{2}$)	Self-Inductance
3AX	r	180	1	0.18	20	0.15756	183.4 μH
	p	152	2	0.18	12	0.02552	226.4 μH
	t	106	2	0.18	9.5	0.02341	2.6 mH
2AXH	p	14	2	0.224	1.1	0.00303	7.4 μH
	t	106	2	0.18	9.5	0.02341	183.3 μH
1AX	t	106	2	0.18	9.5	0.02341	183.33 μH
2AXE	p	152	2	0.18	11.2	0.02353	209.4 μH
	t	106	2	0.18	9.5	0.02341	183.3 μH

**Table 2:** Main parameters of the external layer of the solenoid.

Length *L*	545 mm
Number turns	453
Radius *r*	113.5 mm
On-axis magnetic field $B_{0}$ for 1A of current	0.96421 mT
Equivalent inductance	16.02 mH
Equivalent parallel capacitance	84.1 pF
Equivalent resistance	5.61 Ω

**Table 3:** Impact of the geometrical variations of the solenoid geometry on the on-axis magnetic field with numerical and analytical method.

		Reference	L + 0.5 mm	L − 0.5 mm	r + 0.25 mm	r − 0.25 mm	e = 0.07
Numerical	B(mT)	0.96422	0.96347	0.96497	0.96390	0.96453	0.96440
	ϵ (%)	-	−0.078	0.078	−0.033	0.033	0.018
Analytical	B(mT)	0.96421	0.96346	0.96497	0.96390	0.96453	-
	ϵ (%)	-	−0.078	0.078	−0.033	0.033	-

**Table 4:** Linear regression fit results for the shunt calibration.

Slope	Intercept	$R^{2}$	*p*-Value	Slope Std. Error	Intercept Std. Error
0.100029	5.6 $\times 10^{-5}$	0.999993	2.1 $\times 10^{-69}$	7.1 $\times 10^{-5}$	3.932005 $\times 10^{-5}$

**Table 5:** Fit results for the low-frequency test case.

	Value	σ
$A_{0}$	14.3548	0.0024
*f*	61.99604	0.00027
φ	−0.87750	0.00017

**Table 6:** Different source of errors and their relative percentage impact in measuring the effective area.

Source of Error	ϵ (%)
Shunt	0.03
Solenoid	0.08
Angle misalignment	0.0013
Frequency	0.05
Voltage	0.05

**Table 7:** Number and conditions of applied thermal cycles for each sensor prototype.

	Thermal Cycles, T (°C)						
Sensor	#1	#2	#3	#4	#5	#6	#7
3AXC	160	160	180	180	200	200	200
3AXE	160	180	180	200	200	200	220
3AXF	160	180	180	200	200	200	-

## Data Availability

The data that support the findings of this study are available upon reasonable request from the authors.
